# Quality of life among patients seeking treatment for substance use disorder, as measured with the EQ-5D-3L

**DOI:** 10.1186/s41687-020-00247-0

**Published:** 2020-11-09

**Authors:** Kim Rand, Espen Ajo Arnevik, Espen Walderhaug

**Affiliations:** 1 Health Services Research Unit, Akershus University Hospital, Lørenskog, Norway; 2 Department of Addiction Treatment, Oslo University Hospital, Oslo, Norway

**Keywords:** Drug addiction, Substance use disorder, SUD, EQ-5D, HRQoL

## Abstract

**Purpose:**

There is a need to assess the quality of treatment for Substance Use Disorder (SUD), and document SUD patients’ health-related quality of life (HRQoL). The study aims to describe Norwegian SUD patients’ HRQoL as measured by EQ-5D, compared to a general population sample, and discuss the potential usefulness of the EQ-5D to monitor HRQoL for SUD patients.

**Methods:**

One hundred seventy eight SUD patients (66.3% male) were administered the EQ-5D-3L at treatment start. Patients and general population samples were compared in terms of reported EQ-5D-3L health states, problems by dimension, UK index values, and EQ VAS scores. We investigated specific drug dependence, mental health disorders, sex, age, and education as predictors of EQ-5D-3L values and EQ VAS scores. Anxiety/depression dimension scores were compared to Hopkins symptom Checklist (HSCL-25) scores.

**Results:**

91.6% of the patient sample reported problems on the EQ-5D-3L, with 29.8% reporting extreme problem, compared to 39.8% and 3.0% in the general population sample. Mean index (EQ VAS) score among SUD patients was .59 (59.9) compared to .90 (84.1) in the general population. Regression analyses identified phobic anxiety and cocaine dependence as statistically significant predictors of higher EQ-5D-3L index scores.

**Conclusion:**

SUD patients report substantially reduced HRQoL, as measured using the EQ-5D-3L. The most frequently reported problems were for the anxiety/depression, pain/discomfort, and usual activities dimensions. The EQ-5D may be a useful and practical instrument for monitoring HRQoL in SUD patients.

## Introduction

There is a demand for evidence based treatments and heightened standards in Norway and internationally. National guidelines and standards are developed and implemented (e.g. [1]), but empirical evidence on effect and cost-effectiveness of treatment remains scarce. Thus, parallel to measuring implementation success there is a need for assessment of treatment quality and cost-effectiveness. Individual goals in SUD treatment might cover areas from abstinence to limited use, alleviate co-morbid psychiatric disorders, reduce symptom pressure, facilitate rehabilitation, increase life quality and work ability. There is a need for finding a consensus for which quality indicators are relevant for the formulation of SUD treatment [2].

The general concept of quality of life was initially considered a useful adjunct to traditional concepts of health and functional status. An ideal health assessment would include a measure of the person’s physical health, a measure of physical, social and psychological functioning, and a measure of quality of life [3].

Initially developed to measure health-related quality of life (HRQoL) to measure health benefit in terms of quality-adjusted life-years (QALYs) in health-economic analyses, the EQ-5D is widely used to report on QoL in clinical settings [4]. Being available in a wide range of languages, and for most regular response formats, the instrument is used to report quality of life for a growing number of different populations and settings.

Little is known about the quality of life of patients seeking specialised health treatment for SUD, or the degree to which EQ-5D is a useful tool for measuring quality of life in this patient population. The study had two primary aims: (1) to investigate and describe the self-reported health-related quality of life (HRQoL) as measured by EQ-5D among patients seeking SUD treatment in Norway, and comparison with self-reported health among individuals from the general population; and (2) to discuss the potential of the EQ-5D for use in SUD patients.

## Methods

### Samples

#### SUD patient sample

The sample comprised 178 SUD patients enrolled in the Youth Addiction Treatment Evaluation Project [5]. The SUD patients received treatment in a long-term (3–6 months) residential SUD treatment unit for young adults, targeting patients aged 16 to 28 years, with some leeway. The patients had a mean age of 23.9 years (SD = 3) and 66.3% were men. On average, they had completed 10.8 years (SD = 1.6) of education. Substance dependence was a requirement for admission to treatment. All participants had mental and behavioral disorders due to psychoactive substance use (SUD dependence diagnoses: ICD-10; F10–F19; limited to the FX.2, dependence), and polydrug use was frequent [5]. In addition to SUD diagnoses, the majority of the participants had one or more comorbid diagnoses registered in their electronic patient record (numbers provided in Table 3). Frequent co-morbid disorders include mood and anxiety disorders, posttraumatic stress disorder, personality disorders, and attention-deficit hyperactivity disorder. The data were collected from January 2011 to May 2015 in the Department of Addiction Treatment (Youth), Oslo University Hospital. Treatment of SUD is considered medically necessary, and under the Norwegian public healthcare system, all medically necessary treatments are provided free of charge. All participants were administered the EQ-5D-3L at the beginning of residential SUD treatment.

From patient journal data, we extracted information regarding specific substance-related disorders according to ICD-10 (F10-F19), as well as other clinical diagnoses from the F-chapter. Diagnoses were grouped by main code (e.g. F10.2, indicating alcohol dependence, or F31.x, Bipolar disorder). In addition to these diagnoses, we considered the patients’ age, sex, and level of education (secondary education, 10–13 years; or higher education, > = 14 years).

#### General population sample

Patient responses to the EQ-5D-3L were compared with the responses of individuals below the age of 35 from a general population survey conducted in 2010 in which the EQ-5D-3L was a component. The study from which the norm data is derived is described in detail elsewhere [6].

### Outcome measures

#### EQ-5D

The EQ-5D is a short-form questionnaire designed to capture HRQoL, primarily for use in estimation of QALYs [7, 8]. It is the most widely used instrument for QALY estimation in health-economic analyses [9], and is a preferred or recommended preference-based measure for use in QALY calculation in several countries (e.g. [10, 11]). The EQ-5D instrument consists of two parts: the *descriptive system,* and national *value sets*. The descriptive system refers to a short questionnaire intended for self-administration, in which respondents rate their health along five dimensions of health, followed by a standardized visual analogue scale. National value sets are indexes mapping all possible combinations of responses to the 5 dimensions in the descriptive system to values representing the overall quality of life of the health states. The questionnaire describes health along 5 dimensions: mobility, self-care, usual activities, pain/discomfort, and anxiety/depression. In the original version of the instrument that we used in this study, each dimension can be reported at three different levels, roughly corresponding to: No, moderate, and extreme problems. EQ-5D health states are conventionally referred to using five digit numbers, with each digit representing the level of functioning for the dimensions in the previously presented order, so that state 11111 indicates no problems on any dimension, state 11213 indicates moderate problems with usual activities and extreme anxiety/depression, and 33333 indicates extreme problems with all 5 dimensions. Recently, a new 5-level version has been released. To distinguish between the two versions of the EQ-5D; the original version used in this study is generally referred to as the EQ-5D-3L, and the new version as EQ-5D-5L [12]. In addition to the 5 dimensions, respondents are asked to report their overall quality of life on a thermometer-like vertical visual analogue scale, the EQ VAS, with the top anchor (100) labelled “best imaginable health”, and 0 labelled “worst imaginable health”. The Norwegian translation of the EQ-5D-3L was used in this study.

#### Hopkins symptom checklist

All patients were administered the HSCL-25, followed by the EQ-5D-3L, at the beginning of the residential SUD treatment. The HSCL-25 is a screening instrument extensively used in clinical settings to identify problems with anxiety and depression [13]. It consists of 25 questions, 10 with statements indicative of anxiety and 15 indicative of depression; all with levels corresponding to (1) *Not at all*, (2) *A little*, (3) *Quite a bit*, and (4) *Extremely*. HSCL-25 scores are calculated as the mean of the 25 questions, resulting in a value in the range 1 to 4.

### Analyses

#### Self-reported quality of life

We compared EQ-5D responses taken at the beginning of residential SUD treatment with responses from the general population sample on each of the five dimensions, the EQ-5D-3L index values, and the EQ VAS. There is currently no Norwegian EQ-5D tariff available. Following convention in the field in the absence of national tariffs, the UK EQ-5D-3L tariff [14] was used for index value calculation in both datasets. Mean index and EQ VAS values were compared for all respondents, separately for men and women, and separately by age (<=20 years, 21–25 years, and 26–35 years). Overall distribution of EQ-5D-3L values and EQ VAS scores for SUD patients and the general population respondent sample were presented graphically using density plots, which can be read roughly as continuous histograms, with a total area under the curve of 1. The kernel density was plotted with bandwidth set to 0.5 times the canonical default for nrd0 (i.e. approximately 0.141) and 512 slices per graph. The patient and general population samples’ EQ-5D-3L responses were also juxtaposed in terms of frequently reported EQ-5D health states.

Reported levels of problems on each dimension were compared between SUD patients and general population respondents using Fisher’s exact test for proportions.

As a limited indicator of convergent validity with HSCL-25, EQ-5D-3L index values and scores on the anxiety/depression dimension were correlated with HSCL-25 total scores, and the three combined graphically.

#### Predictors of self-reported quality of life

We used a two-stage multivariate linear regression to describe how EQ-5D index values and EQ VAS values were associated to diagnostic and demographic patient indicators. In the first stage, each potential candidate parameter was used to predict index or EQ VAS values in a bivariate model. Statistically significant variables (critical *p* value < 0.05) from these tests were included in a multivariate model. Breush-Pagan and non-constant variance tests were used to test for heteroscedasticity, in which case the tests were rerun with Box-Cox transformed dependent variables as a sensitivity analysis.

#### Critical *p*-value and statistical packages

We used a critical level of ≤ .05 for statistical significance. All analyses were performed in the R statistical package version 3.2.3 [15]. Graphs were made using the ggplot2 package [16].

## Results

One hundred seventy eight SUD respondents aged 17–33 years (mean 23.8 years, SD 3.2) completed the EQ-5D-3L questionnaire at the beginning of residential SUD treatment. Age and sex of the study participants and the 400 respondents (mean age 27.4 years, SD 5.3) in the general population sample can be found in Table 1. Further information regarding the study from which the general population sample derives can be found in [6].

**Table 1:** By sample, age, and sex: number of respondents (%); mean (SD) index values; and mean (SD) EQ VAS scores

Age	Substance abuse patients			General population sample		
	Male	Female	All	Male	Female	All
**Number (%) of patients/respondents**						
All	118 (100.0%)	60 (100.0%)	178 (100.0%)	167 (100.0%)	233 (100.0%)	400 (100.0%)
< −- 20	18 (15.3%)	8 (13.3%)	26 (14.6%)	23 (13.8%)	35 (15.0%)	58 (14.5%)
21–25	66 (55.9%)	36 (60.0%)	102 (57.3%)	40 (24.0%)	59 (25.3%)	99 (24.8%)
26–35	34 (28.8%)	16 (26.7%)	50 (28.1%)	104 (62.3%)	139 (59.7%)	243 (60.8%)
**EQ-5D-3L value, UK value set (SD)**						
All	0.62 (0.29)	0.53 (0.33)	0.59 (0.31)	0.92 (0.14)	0.88 (0.18)	0.90 (0.16)
< −- 20	0.71 (0.29)	0.40 (0.39)	0.61 (0.35)	0.95 (0.09)	0.87 (0.16)	0.90 (0.14)
21–25	0.59 (0.28)	0.50 (0.32)	0.56 (0.30)	0.92 (0.10)	0.91 (0.12)	0.92 (0.12)
26–35	0.61 (0.32)	0.65 (0.30)	0.62 (0.31)	0.91 (0.16)	0.86 (0.20)	0.88 (0.18)
**EQ VAS score (SD)**						
All	61.44 (21.94)	56.75 (20.52)	59.86 (21.53)	84.13 (11.78)	84.10 (15.54)	84.11 (14.08)
< −- 20	62.22 (20.16)	52.50 (21.88)	59.23 (20.77)	86.91 (10.33)	86.37 (15.22)	86.59 (13.40)
21–25	61.21 (20.29)	57.08 (20.26)	59.75 (20.28)	84.05 (10.30)	86.25 (12.30)	85.36 (11.53)
26–35	61.47 (26.18)	58.12 (21.52)	60.40 (24.62)	83.54 (12.59)	82.62 (16.73)	83.01 (15.07)

Table 2 describes the EQ-5D-3L health states self-assigned by the SUD respondents and their general population counterparts. 60.2% of the general population sample respondents described themselves as being in state 11111 (no health problems on any dimension). In contrast, only 15 of the 178 SUD patients, 8.4%, reported state 11111. Three percent of the general population sample reported extreme problems on any dimension, compared to 29.8% of the SUD patients. The general population sample self-assigned a total of 24 different EQ-5D health states, of which the most frequent 6 (states 11111, 11112, 11121, 11122, 11221, and 11222) accounted for 91.5% of the respondents. The SUD patients self-assigned 36 different health states, and the 6 most frequent states (11,111, 11,222, 11,122, 11,112, 11,212, and 21,223) accounted for 54.5% of the patients.

**Table 2:** Self-reported EQ-5D-3L health state characteristics by SUD patients and Norwegian general population samples, n (%)

**A. Frequently reported health states by group**									
		SUD patients					Norwegian general population		
State	Value	Male	Female	All	State	Value	Male	Female	All
All		118 (100.0%)	60 (100.0%)	178 (100.0%)	All		167 (100.0%)	233 (100.0%)	400 (100.0%)
11222	0.689	21 (17.8%)	9 (15%)	30 (16.9%)	11111	1.000	110 (65.9%)	131 (56.2%)	241 (60.3%)
11122	0.725	11 (9.3%)	5 (8.3%)	16 (9%)	11112	0.848	21 (12.6%)	25 (10.7%)	46 (11.5%)
11111	1.000	12 (10.2%)	3 (5%)	15 (8.4%)	11121	0.796	14 (8.4%)	18 (7.7%)	32 (8%)
11112	0.848	11 (9.3%)	3 (5%)	14 (7.9%)	11122	0.725	12 (7.2%)	17 (7.3%)	29 (7.3%)
11212	0.812	6 (5.1%)	7 (11.7%)	13 (7.3%)	11221	0.760	2 (1.2%)	7 (3%)	9 (2.3%)
21223	0.186	6 (5.1%)	3 (5%)	9 (5.1%)	11222	0.689	2 (1.2%)	7 (3%)	9 (2.3%)
30 other	avg: 0.405	51 (43.2%)	30 (50%)	81 (45.5%)	18 other	avg: 0.544	6 (3.6%)	28 (12%)	34 (8.5%)
**B. Self-reported problems on EQ-5D dimensions** ^*^									
		SUD patients					Norwegian general population		
		Male	Female	All			Male	Female	All
**All**		118 (100.0%)	60 (100.0%)	178 (100.0%)	**All**		167 (100.0%)	233 (100.0%)	400 (100.0%)
**Mobility**					**Mobility**				
No problems		85 (72.0%)	43 (71.7%)	128 (71.9%)	No problems		162 (97.0%)	215 (92.3%)	377 (94.2%)
Moderate problems		33 (28.0%)	17 (28.3%)	50 (28.1%)	Moderate problems		5 (3.0%)	18 (7.7%)	23 (5.8%)
Extreme problems		–	–	–	Extreme problems		–	–	–
**Self-care**					**Self-care**				
No problems		107 (90.7%)	53 (88.3%)	160 (89.9%)	No problems		167 (100.0%)	231 (99.1%)	398 (99.5%)
Moderate problems		11 (9.3%)	6 (10.0%)	17 (9.6%)	Moderate problems		–	2 (0.9%)	2 (0.5%)
Extreme problems		–	- 1 (1.7%)	1 (0.6%)	Extreme problems		–	–	–
**Usual activities**					**Usual activities**				
No problems		49 (41.5%)	17 (28.3%)	66 (37.1%)	No problems		159 (95.2%)	202 (86.7%)	361 (90.2%)
Moderate problems		65 (55.1%)	41 (68.3%)	106 (59.6%)	Moderate problems		8 (4.8%)	29 (12.4%)	37 (9.2%)
Extreme problems		4 (3.4%)	2 (3.3%)	6 (3.4%)	Extreme problems		–	2 (0.9%)	2 (0.5%)
**Pain/discomfort**					**Pain/discomfort**				
No problems		42 (35.6%)	20 (33.3%)	62 (34.8%)	No problems		133 (79.6%)	163 (70.0%)	296 (74.0%)
Moderate problems		67 (56.8%)	33 (55.0%)	100 (56.2%)	Moderate problems		32 (19.2%)	67 (28.8%)	99 (24.8%)
Extreme problems		9 (7.6%)	7 (11.7%)	16 (9.0%)	Extreme problems		2 (1.2%)	3 (1.3%)	5 (1.2%)
**Anxiety/depression**					**Anxiety/depression**				
No problems		23 (19.5%)	7 (11.7%)	30 (16.9%)	No problems		130 (77.8%)	165 (70.8%)	295 (73.8%)
Moderate problems		70 (59.3%)	32 (53.3%)	102 (57.3%)	Moderate problems		36 (21.6%)	63 (27.0%)	99 (24.8%)
Extreme problems		25 (21.2%)	21 (35.0%)	46 (25.8%)	Extreme problems		1 (0.6%)	5 (2.1%)	6 (1.5%)

^*^Fisher’s Exact Test comparing SUD and corresponding general population *p* < 0.001 for all groups and subgroups

*SUD* Substance Use Disorder

Broken down by dimension and level, the SUD group were statistically significantly more likely to report problems on all five dimensions (Fisher’s exact test *p* < 0.001 for all dimensions, for both sexes separately and combined), with the most substantial difference for usual activities, pain/discomfort, and anxiety/depression (see Table 2).

**Fig. 1:**
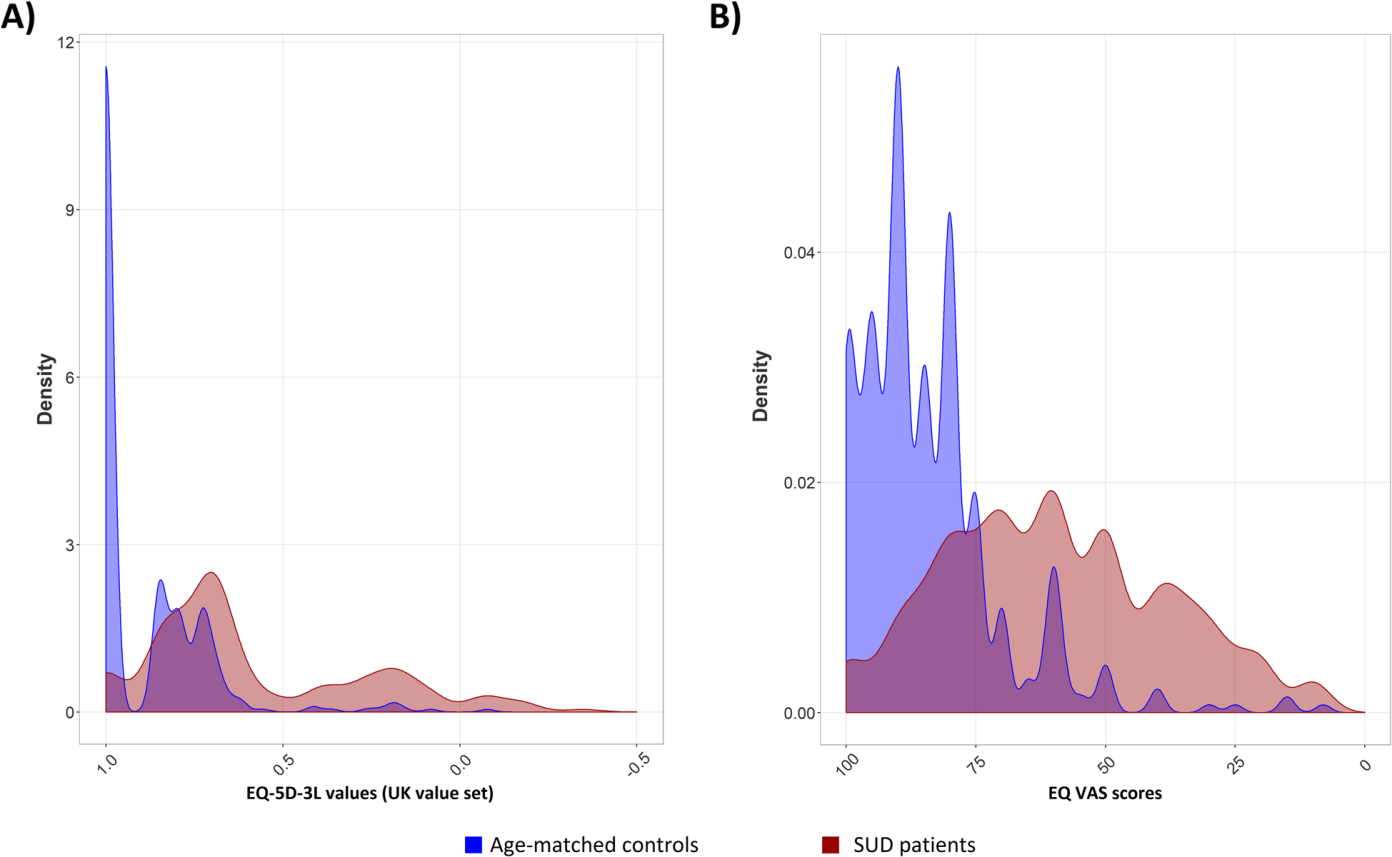
Density plots for SUD patients (red) and general population respondents (blue), separately for (**a**) EQ-5D-3L index scores, and (**b**) EQ VAS scores

The SUD sample’s EQ-5D-3L index values and EQ-VAS scores at the beginning of treatment were substantially reduced compared to the general population sample; both for the sample as a whole, and all sub-groups split by sex and age (see lower parts of Table 1). Where the general population sample displayed a mean index (EQ VAS) value of .90 (84.1), the corresponding SUD mean was 0.59 (59.9). The SUD distributions of index and EQ VAS values were shifted downwards substantially from their general population counterparts (see Fig. 1), indicating substantially reduced health-related quality of life.

The bivariate regression models included age, sex, education, and diagnostic codes. Heteroscedasticity as measured by Breush-Pagan tests and non-constant variance tests were confirmed in most of the regression analyses. However, Box-Cox-transformation of the dependent variables did not result in changes of note for interpretation, with no changes of direction or statistical significance observed. Consequently, we report the OLS results here (see Table 3). This procedure identified sex, F14.2 (cocaine dependence) and F40.x (phobic anxiety) as statistically significant candidate predictors for predicting EQ-5D-3L values and EQ VAS scores. When collectively entered in multiple regression models, cocaine dependence (F14.2) was associated with statistically significantly higher EQ-5D-3L values (0.25, *p* = 0.0089) and EQ VAS scores (15.5 points, *p* = 0.0121). Phobic anxiety (F40.x) was associated with higher EQ-5D-3L values (0.353, *p* = 0.0173). Phobic anxiety was non-significantly related to EQ VAS score, and the association with sex was not statistically significant in the multivariate regression models.

**Table 3:** Regressions predicting EQ-5D-3L index value, EQ-VAS, and HSCL-25 scores

**A) Univariate regressions**							
		Dependent variables					
		EQ-5D-3L value		EQ VAS score		HSCL-25 score	
Coefficients	*n*	Estimate	*p*	Estimate	*p*	Estimate	*p*
Demographics							
Sex (dummy for female)	60	−0.1001	0.0396*	−5.6470	0.0965	0.3950	0.0000*
Secondary education (10–13 years)	123	− 0.0301	0.5872	5.0868	0.1946	0.1010	0.3081
Higher education (> = 14 years)	6	0.0431	0.7494	2.6065	0.7847	0.0004	0.9988
Age (years)	178	0.0002	0.9835	0.3630	0.4754	−0.0099	0.4432
Drug-related diagnoses (ICD-10)							
F10.2 Alcohol	57	−0.0294	0.7231	3.4368	0.5060	0.1007	0.4952
F11.2 Opioids	57	−0.0418	0.6093	3.0238	0.5533	0.2383	0.0993
F12.2 Cannabis	105	−0.0627	0.4276	−7.7067	0.1152	0.1950	0.1639
F13.2 Sedatives	89	0.0190	0.8056	1.2811	0.7897	0.0239	0.8620
F14.2 Cocaine	26	0.2277	0.0214*	14.5098	0.0186*	−0.4539	0.0097*
F15.2 Other stimulants	78	0.1366	0.0692	6.0409	0.1998	−0.1518	0.2602
F19.2 Mixed drug abuse	40	−0.0137	0.8807	9.5541	0.0913	0.0811	0.6192
Clinical diagnoses (ICD-10)							
F31.x Bipolar	7	0.1687	0.4772	22.1364	0.1322	−0.1713	0.6857
F32.x Major depression, single	20	0.1929	0.1449	1.9571	0.8137	−0.3154	0.1812
F33.x Major depression, recurring	30	0.0392	0.7290	2.2531	0.7492	0.0049	0.9804
F40.x Phobic anxiety	23	0.3088	0.0428*	10.8077	0.2601	−0.5100	0.0609
F41.x Other anxiety	14	0.1958	0.1611	5.6210	0.5210	−0.2912	0.2431
F43.1 PTSD	27	0.1417	0.2510	4.5545	0.5554	−0.1479	0.5025
F60.x Personality disorder	22	−0.0939	0.4220	−1.5708	0.8298	0.0605	0.7719
F90.x ADHD	12	0.0736	0.6338	3.1346	0.7449	0.0688	0.8028
**B) Multivariate regressions**							
		EQ-5D-3L value		EQ VAS score		HSCL-25 score	
Coefficients		Estimate	*p*	Estimate	*p*	Estimate	*p*
(Intercept)	178	0.5892	0.0000*	61.4733	0.0000*	2.0964	0.0000*
Female	60	−0.1340	0.1083	−8.9640	0.0925	0.4198	0.0053*
F14.2 Cocaine	26	0.2537	0.0089*	15.5018	0.0121*	−0.4978	0.0039*
F40.x Phobic Anxiety	23	0.3528	0.0173	13.4907	0.1484	−0.5962	0.0228*

*HSCL* Hopkins Symptom Checklist, *ICD-10* International Classification of Diseases version 10, *PTSD* Post-Traumatic Stress Disorder, *ADHD* Attention Deficit Hyperactivity Disorder, * indicates statistical significance (*p* <=0.05)

**Fig. 2:**
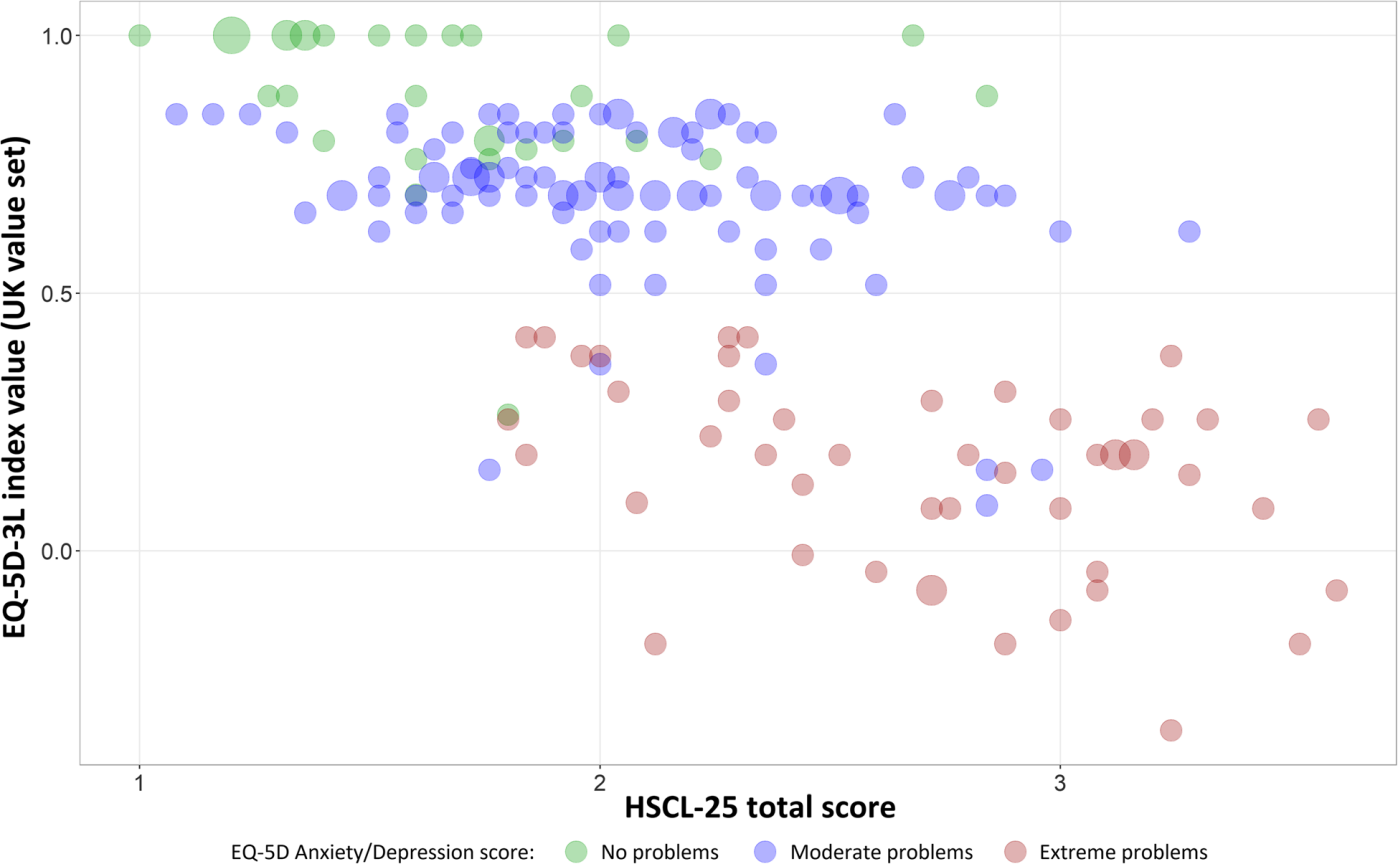
Observed self-reported EQ-5D-3L index scores (UK value set) over corresponding HSCL-25 total scores in the SUD population. Circle area proportional to number of participants assigning particular combination. Circle colors represent self-rating on the Anxiety/Depression dimension of the EQ-5D-3L: green, no problems; blue, moderate problems; red, extreme problems

As expected, there was a statistically significant negative correlation between EQ-5D-3L values and corresponding HSCL-25 mean value (Pearson’s R = − 0.671, *p* < 0.0001), and between HSCL-25 mean and corresponding response to the anxiety/depression dimension of the EQ-5D-3L (Pearson’s R = 0.613, *p* < 0.0001, see Fig. 2).

## Discussion

The main finding was that residential SUD patients reported having substantial problems influencing their health-related quality of life as measured using the EQ-5D-3L, with mean index value of.59 at the beginning of treatment, compared to.90 for the general population sample. Where more than half of the general population sample reported no problems on any dimension, only 8.4% of the patients did so. Conversely, more than 30% of the patients reported having at least one dimension with extreme problems, compared to 3.0% of the general population respondents. For comparison to the mean EQ-5D-3L score found in this study, Saarni et al. [17] reported mean QoL/HRQoL scores based on a sample of Finnish people with various mental health problems, using the EQ-5D-3L and the UK value set (as in this study), including schizophrenia (.715), schizoaffective disorder (.681), major depression with psychotic features (.707), and other psychotic disorders (.639). Our observed mean value for SUD patients is in the same range as Saarni’s reported mean values for other psychotic disorders, and significantly below those reported for e.g. Schizophrenia (*p* = .0012, not adjusted for differences in e.g. age). More recently, Olesen and colleagues [18] reported mean EQ-5D-3L values for various chronic conditions in Denmark, the lowest of which were cerebral thrombosis (.621) and angina (.648). They also report decreasing values with increasing number of chronic disorders, such that patients with five or more conditions had a mean EQ-5D-3L value of .597. If we consider somatic patient groups for which substantial studies of QoL have been performed, mean EQ-5D-3L values for cancer patients tend to be reported above .6, and mean EQ VAS between .55 and .8 (See e.g., [19]). These comparisons should not be interpreted as measures of the relative misfortune of these patient groups, but rather as an indication that the SUD patients in this study report experiencing substantially impaired health and HRQoL. In addition to the heightened mortality of SUD patients, this underlines the importance of identifying successful strategies and treatment options for this patient group.

We also found that the most commonly reported problems were with anxiety/depression, pain/discomfort, and usual activities. Reflecting the comparatively good physical health of the patient group, none of the patients reported being at the worst level on mobility, only one for self-care, and 7 (4%) for usual activities. The responses to the anxiety/depression scale, arguably the most relevant subscale for this patient group, were corroborated by corresponding scores on the HSCL-25, with more than 37% variance explained by the single EQ-5D-3L item. The responses to the EQ VAS were similarly reduced, indicating that the reported health problems were experienced as being detrimental to the patients’ quality of life.

These results suggest that the EQ-5D is sensitive to dimensions of health for which SUD patients experience health problems. Furthermore, the observed reductions in HRQoL implies substantial potential QALY-gains if the patient group were to be successfully treated. In cost/QALY analyses, this would allow for relatively large investments for treatment programs improving the quality of life of SUD patients. However, while observation of reduced QoL at the initiation of treatment suggest that the EQ-5D could be a useful tool in clinical practice, there is call for research investigating the sensitivity of the EQ-5D to changes in the QoL of SUD patients. Additionally, the EQ-5D-3L used in this study uses very wide categories within each dimension, with levels corresponding roughly to “no”, “moderate”, and “extreme” problems. Future research could benefit from the newer EQ-5D-5L, with 5 levels for each dimension, ideally allowing for greater sensitivity to smaller changes in health.

The EQ-5D is a generic instrument, intended to capture broad aspects of health that are of importance to a wide range of patients and conditions, with a minimal number of questions. As such, it is extremely reductionist, covering only 5 dimensions of health, each with a single item. Given this design, the EQ-5D is unlikely to provide a comprehensive description of the symptoms and problems experienced by any particular patient group. More importantly, for certain patient groups, important issues fall outside the scope of the questionnaire. For instance, Saarni and colleagues [17] report that the EQ-5D values of patients with delusional or bipolar 1 disorders were not statistically significantly different from the general population. If we accept that the QoL of these patient groups is likely to be impaired in reality, this suggests that the EQ-5D does not adequately capture the areas in which these patient groups experience problems. For SUD patients, issues such as stigma and craving might not be fully captured, though both may be partially covered by anxiety/depression and pain/discomfort. Given the brevity of the EQ-5D, it is likely insufficient for clinical monitoring of SUD patients used in isolation. However, this study suggests that the EQ-5D may be suited to capture a wide range of relevant problems experienced by SUD patients, particularly the dimensions anxiety/depression, pain/discomfort, and self-care. As such, the EQ-5D, including the EQ VAS, could potentially form the backbone of a brief, low-cost battery of questions suitable for use in routine monitoring of SUD patients and their symptom.

A longitudinal cohort study found that SUD patients who successfully quit substance use for 1 year showed improved satisfaction with life and reduced psychological distress, compared to SUD patients that relapsed and control participants [20]. The patient population in the Hagen study is similar to the SUD patients described here, and it is reasonable to assume that successful SUD treatment followed by drug abstinence would lead to improved HRQoL, which would hopefully be reflected in EQ-5D values. However, a recommendation to use the EQ-5D in monitoring HRQoL in clinical SUD settings should be considered experimental until the sensitivity to change in this population has been established.

The regression analyses indicate that SUD patients using cocaine report comparatively higher self-reported health and HRQoL than users of other substances. The reasons for this are not apparent, but it is plausible that there are systematic differences between users of different substances in terms of wealth and socio-economic status, with accompanying differences in terms of non-drug-related habits and health, all of which could influence mean reported HRQoL. The analyses also revealed a statistically significant association between phobic anxiety and higher EQ-5D-3L values. Considering that the anxiety/depression dimension is one of the drivers of low EQ-5D-3L values in the SUD sample, this is counterintuitive. However, phobic anxiety is characterized by heightened anxiety levels in response to particular stimuli, which one may hope are not present while undergoing treatment. As such, we may speculate that individuals with phobic anxiety could experience lower anxiety levels while undergoing treatment. Similar effects could be in play for the counter-intuitive statistically non-significant positive point estimates observed for e.g. PTSD and major depression, though these could simply reflect individual variation in responses. Furthermore, the absence of previous diagnoses of e.g. depression could reflect fewer previous contacts with the health care system, rather than better current mental health.

There is a paucity of research on HRQoL and instruments such as the EQ-5D in SUD patients, though a few studies have reported on particular subgroups. Günther and colleagues found that the EQ-5D-3L was less responsive than other tested instruments (GAF, WHOQOL-BREF, and HoNOS) in alcohol dependent patients [21]. van der Zanden and colleagues reported on the suitability of EQ-5D-3L in a sample of 430 patients in Dutch heroin treatment trials, and concluded that the instrument appears suited for this population [22]. Dalen et al. reports on a 2015 study of 365 SUD patients in an outpatient setting in northern Norway, where EQ-5D-3L was included. The results described were limited to proportions of patients reporting problems on mobility (25%, 28.1% in our study), usual activities (49%, 63% in our study), pain/discomfort (68%, 65.2% in our study) and anxiety/depression (60%, 83.1% in our study); and mean EQ VAS score (60, compared to 59.86 in our study) [23]. The reported numbers are strikingly well-aligned, though the sample in our study report somewhat more problems.

One of the major obstacles to successful treatment of SUD patients is drop-out from treatment. Though the rate varies by the definition used to define drop-out, reported rates of 50% are common, and useful methods for prediction and prevention of drop-out are elusive [24]. A research question of potential interest is whether HRQoL can be used to predict drop-out. This requires careful consideration, as it is possible that patients experiencing good quality of life will be less motivated to remain in treatment, meaning that high HRQoL scores could be an indicator of risk. Similarly, low HRQoL scores could be indicative of poor functioning, which could also be a risk factor.

This study is limited in many ways. The patient sample is relatively small, and covers a narrow range of ages, with all participants below the age of 34. Generalizations beyond this age range should be made with care. Similarly, the characteristics of SUD patients could vary between countries and regions, and the problems experienced by SUD patients could vary between societies, health care, and social security systems. Norway is a comparatively wealthy country with low wealth inequality and universal health care, characteristics generally indicative of good population health. Lack of universal health care would be expected to negatively impact the health, and presumably HRQoL, of SUD patients. At the same time, it is possible that failing to meet societal expectations in a country with generally high levels of affluence may result in heightened stigma. Investigations of HRQoL in SUD patients in other countries are called for in order to build a foundation for making generalizations beyond wealthy northern European countries. As noted, the comparatively higher HRQoL reported by users of cocaine remains unexplained, and there may be systematic differences between cocaine users and other SUD patients related to e.g. socio-economic status, markers of which were not included in this study.

## Conclusions

Residential SUD patients reported severely impaired HRQoL on the EQ-5D-3L, with mean index values below previously reported means for patients with serious mental health issues (e.g. schizophrenia, psychosis), chronic somatic diseases, and cancer. This highlights the importance of developing or identifying appropriate treatment options for SUD patients. The most commonly impaired dimensions were anxiety/depression, pain/discomfort, and self-care. The index values and the anxiety/depression score alone both display high correlation with HSCL-25 total scores, indicating good coverage of these constructs. In addition to covering a wide range of problems of relevance to SUD patients at very low burden to the respondent, the EQ-5D allows for calculation of QALYs in cost-effectiveness studies of SUD treatment programs. Considering the low EQ-5D-3L values observed in this study, there is substantial potential for documented QALY gains in the SUD population. There is call for investigation of the sensitivity of the EQ-5D to changes in SUD patients’ HRQoL, for studies using the newer 5-level version of the EQ-5D, the predictive value of EQ-5D for SUD outcomes, and for studies of SUD patients in age groups and countries different from Norway. Given future demonstration of sensitivity to changes in health and HRQoL, the EQ-5D may be a promising starting point for a brief battery of questions used in routine monitoring of SUD patients.

## Data Availability

The datasets analysed during the current study are not publicly available due to Norwegian privacy laws. An anonymized extract containing EQ-5D-3L health states, corresponding index values, and EQ VAS scores, sex, and age group can be obtained from the corresponding author on reasonable request.

## Abbreviations

EQ VAS: Thermometer-like VAS presented alongside EQ-5D questionnaire
EQ-5D: Proper name of 5-dimensional HRQoL instruments owned by EuroQol Group
EQ-5D-3L: 3-level version of EQ-5D
EQ-5D-5L: 5-level version of EQ-5D
HRQoL: Health-related quality of life
HSCL-25: Hopkins Symptom Checklist, 25-item questionnaire
ICD-10: International Classification of Diseases, version 10
QALY: Quality-adjusted life-years
QoL: Quality of Life
SD: Standard Deviation
SUD: Substance Use Disorder
VAS: Visual Analogue Scale
